# Conformal Radiation-Type Programmable Metasurface for Agile Millimeter-Wave Orbital Angular Momentum Generation

**DOI:** 10.34133/research.0631

**Published:** 2025-03-14

**Authors:** Anjie Cao, Tao Ni, Yuhua Chen, Longpan Wang, Zhenfei Li, Xudong Bai, Fuli Zhang, Zhansheng Chen

**Affiliations:** ^1^ Shanghai Institute of Satellite Engineering, Shanghai 201109, China.; ^2^ Northwestern Polytechnical University, Xi’an 710129, China.; ^3^ Shanghai Academy of Spaceflight Technology, Shanghai 201109, China.

## Abstract

Since the scarcity of bandwidth resources has become increasingly critical in modern communication systems, orbital angular momentum (OAM) with a higher degree of freedom in information modulation has become a promising solution to alleviate the shortage of spectrum resources. Consequently, the integration of OAM with millimeter-wave technology has emerged as a focal point in next-generation communication research. Recently, programmable metasurfaces have gained considerable attention as essential devices for OAM generation due to real-time tunability, but their profiles are relatively high as a result of the external feed source. This paper proposes a conformal radiation-type programmable metasurface operating in the millimeter-wave band. By employing a series–parallel hybrid feed network to replace conventional external feed sources, the overall profile of the metasurface system can be reduced to less than 0.1*λ*. Furthermore, the proposed innovation design could also achieve a conformal cross-shaped architecture, which is ultraportable and very effective in integrating with the front ends of satellites or aircraft and eliminating issues such as feed source blockage as well as energy spillover losses in conventional metasurfaces. The proposed metasurface could achieve a realized gain of 22.54 dB with an aperture efficiency of 21.75%, thus generating high-purity OAM waves with topological charges of *l* = 0, *l* = +1, *l* = +2, and *l* = +3. Additionally, by incorporating beam scanning techniques, OAM waves could be deflected to accommodate scenarios with moving receivers, demonstrating substantial potential for future high-speed wireless communication applications.

## Introduction

With the rapid development of modern communication technology, bandwidth resources have become increasingly scarce, and the issue of electromagnetic interference is more prominent than ever [1]. As a result, antennas with high-speed and wide bands have become indispensable in modern wireless communication. Traditional 5G communication technology relies on increasing bandwidths to achieve higher speeds, but the limited bandwidth resources present a bottleneck that cannot be easily overcome [2,3]. Therefore, in the era of 5G-A or 6G, the combination of orbital angular momentum (OAM) with millimeter-wave (mmWave) frequencies offers the potential for even higher transmission rates on top of the large bandwidth [4,5]. As early as the 5G era, the integration of OAM and mmWave technology enabled the reuse of multi-input multi-output technology, thus achieving 16-Gbps mmWave communication [6]. The wavefront of a vortex wave exhibits a spiral shape, carrying OAM, with its phase distribution function containing the term exp(*ilθ*), which is related to the azimuthal angle *θ* [7,8]. This characteristic provides a new degree of freedom for information transmission, allowing OAM waves to transmit multiple signal streams without additional spectrum resources [9]. Compared to traditional electromagnetic waves, OAM waves offer a higher degree of freedom in information modulation, which helps to alleviate the scarcity of spectrum resources [10–13]. All these research findings indicate that OAM associated with mmWave or even terahertz frequencies could hold great potential for significantly increasing communication capacity in future generations of wireless communication, making it a promising solution for overcoming bandwidth limitations [14–16].

OAM beams can be generated through various methods, such as directly using lasers, although this approach is costly [17,18]. Optical fibers can also produce vortex waves but with significant losses [19,20]. Vortex beams stimulated by traditional large-scale phased arrays require radio-frequency modules for each element, which contain a pair of T/R modules and numerous phase shifters, thus greatly increasing the overall complexity and cost [21]. In contrast, programmable metasurfaces could greatly minimize associated expenses for generating OAM beams [22–24]. The novel programmable metasurfaces, with subwavelength profiles and real-time control of elements via PIN diodes using a field-programmable gate array (FPGA), enable dynamic electromagnetic wave generation, positioning them as strong contenders for efficiently producing high-purity OAM waves [25–28]. Currently, numerous studies utilize reflective or transmissive programmable metasurfaces to generate OAM electromagnetic waves [29–33]. For example, in 2022, a reflective programmable metasurface with PIN diodes controlled by an FPGA was introduced to generate multimode high-purity OAM waves in the Ka band [34]. Later, an analytical procedure for designing OAM multiplexers with the capability of generating multiplexed beams was proposed based on a reflective programmable metasurface [35]. However, reflective metasurfaces suffer from severe feed blockage, thereby negatively impacting OAM wave generation, which led to the development of transmissive metasurfaces [36,37]. In 2020, an X-band transmissive digital metasurface was designed for efficiently stimulating dynamic OAM wave beams with high mode purity [38]. Thereafter, a Huygens metasurface based on a dual-layer metal structure was proposed to generate highly efficient dual-polarized OAM vortex waves at 28 GHz [39]. Recently, Qin et al. [40] successfully designed a transmissive metasurface with a uniform circular array feed source, achieving the conversion of OAM from lower-order to higher-order modes through secondary phase regulation of the vortex wavefront. However, traditional reflective or transmissive metasurfaces require feed sources placed at a certain distance, significantly increasing the overall profile of the system, as well as making it unsuitable for current low-profile antenna requirements [41]. To overcome this issue, research on radiation-type metasurfaces has emerged. In 2022, a metasurface array antenna integrating impedance transformer circuits as well as filters and a hybrid series–parallel feeding network was designed, which could achieve dynamic beam scanning and multimode OAM generation in the C band [42]. However, the filtering coupling unit structure was too large and very difficult to implant into high-frequency mmWave metasurfaces; moreover, the square-shaped metasurface architecture is unable to adapt to the conformal design of special shapes based on practical scenarios. Therefore, there is an urgent need for a mmWave metasurface system that maintains high performance while offering a low profile and excellent conformability.

In light of the above challenges, this paper presents a conformal radiation-type programmable metasurface for mmWave OAM generation, which could achieve a significant reduction of the overall profile through utilizing a hybrid series–parallel feeding network to replace the traditional external feed source. Furthermore, the proposed innovation design could also achieve conformal cross-shaped architecture, which is very effective in integrating with the front ends of satellites or aircraft and eliminating issues such as feed source blockage and energy spillover losses in conventional metasurfaces. Based on the proposed design, the metasurface prototype was fabricated, and its application for generating high-purity multimode mmWave OAM beams was verified both numerically and experimentally. The proposed metasurface could obtain a wide operation bandwidth of over 10% and a measured gain of 22.54 dBi with an aperture efficiency of over 21.75% while successfully exciting 4 high-purity OAM wave modes, namely, *l* = 0, *l* = +1, *l* = +2, and *l* = +3. The proposed radiation-type programmable metasurface demonstrates significant potential for future space-based satellite communication and next-generation wireless communication technologies.

## Results and Discussion

**Fig. 1.**
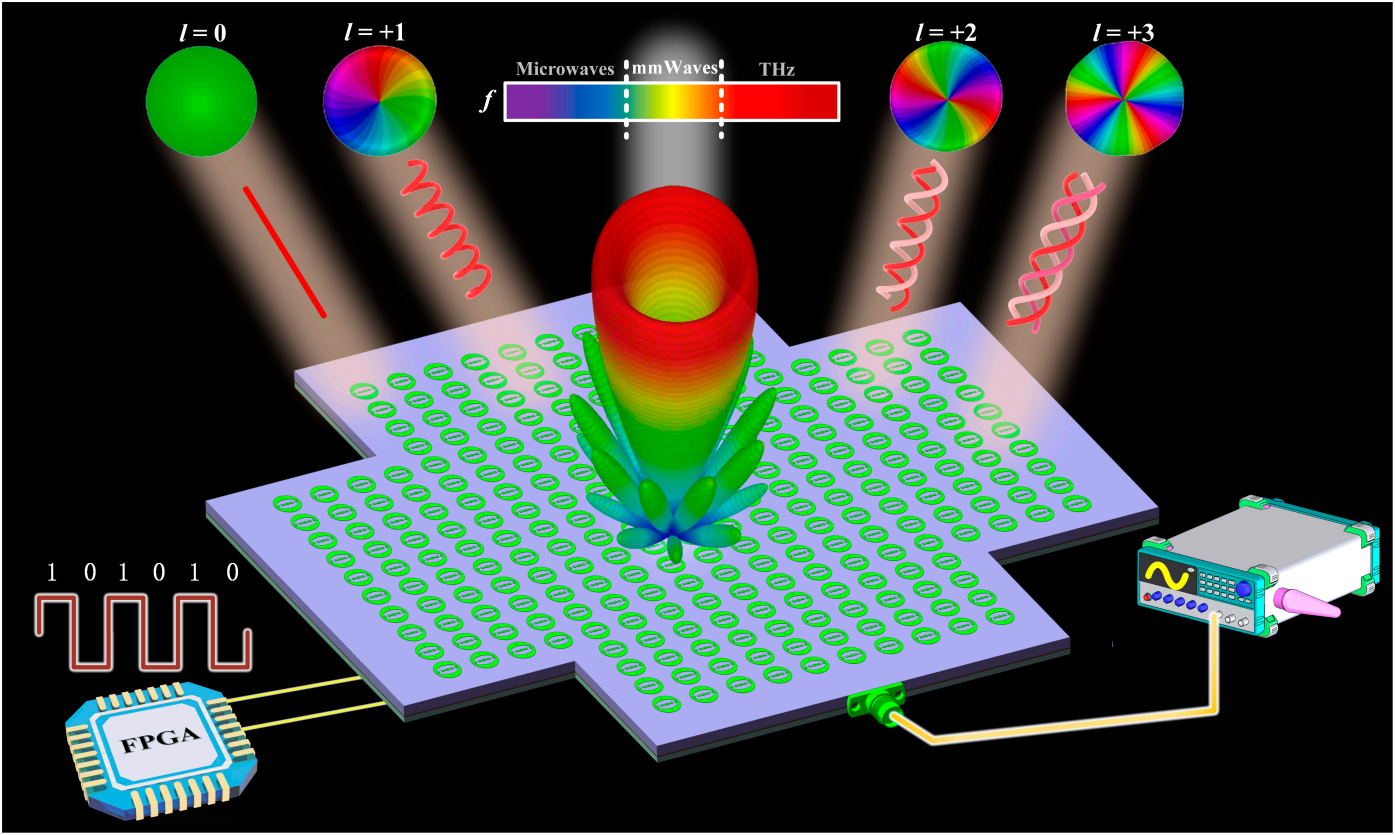
Schematic diagram of the conformal radiation-type metasurface for multimode millimeter-wave (mmWave) orbital angular momentum (OAM) beam generation. FPGA, field-programmable gate array.

Figure 1 illustrates the multimode mmWave OAM beams generated by the conformal radiation-type programmable metasurface. Unlike traditional reflective or transmissive metasurfaces that rely on an external space-feed source, this novel design could eliminate the need for external feeding required by its traditional counterpart by directly connecting the signal source to the metasurface via an SMA connector, thus significantly reducing the system overall profile. The feeding is accomplished through a series–parallel hybrid feed network integrated on the backside of the metasurface array. Additionally, an FPGA is employed to apply high and low voltage levels to control the on/off states of the PIN diodes integrated in the metasurface, thus enabling 0 and π encoding for each unit. This configuration successfully excites high-purity mmWave OAM beams with modes of *l* = 0, *l* = +1, *l* = +2, and *l* = +3.

### Metasurface unit design

**Fig. 2.**
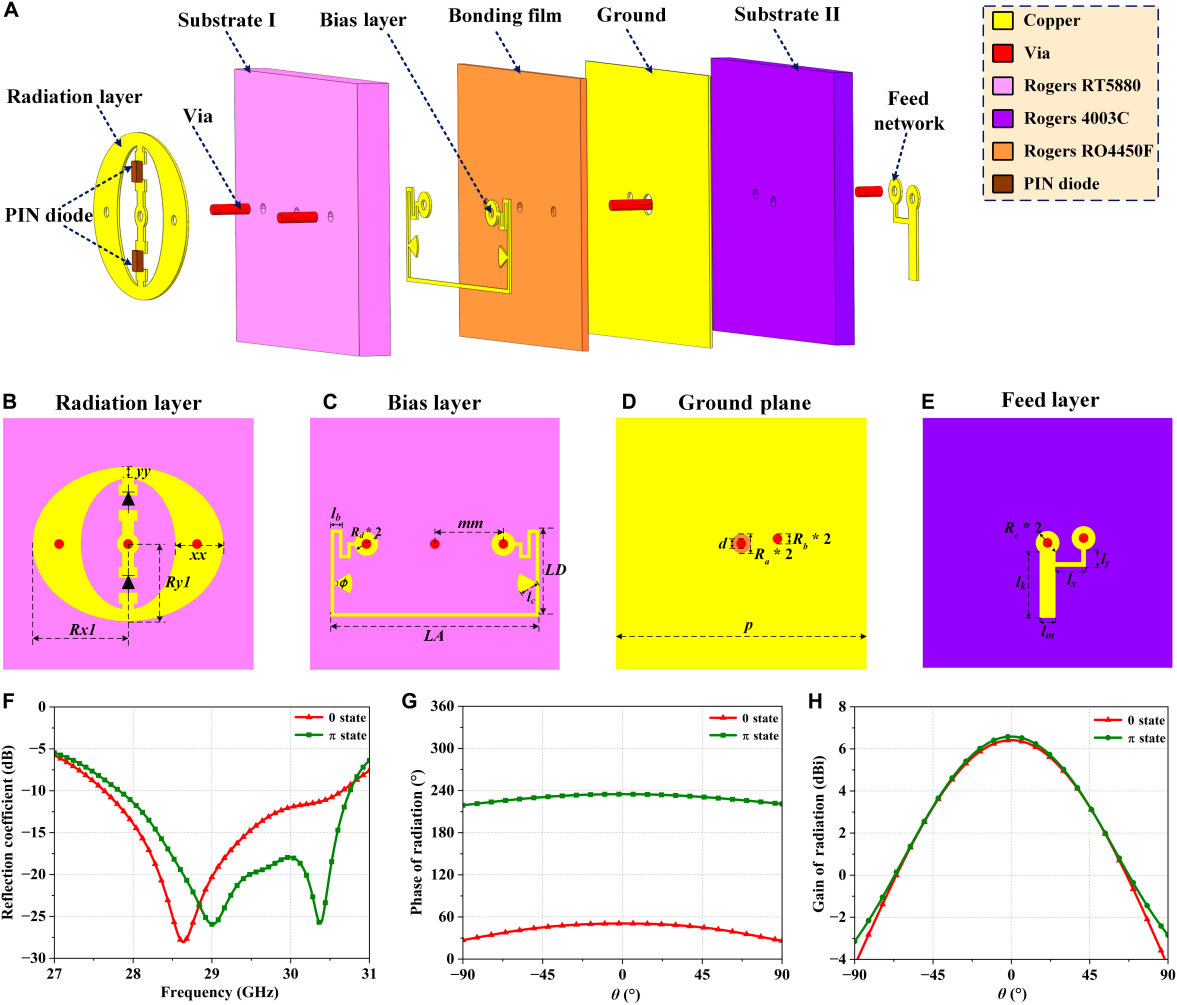
Structure of the mmWave radiation-type programmable metasurface unit. (A) Exploded view of the metasurface unit. (B) Top radiation layer. (C) Middle bias layer. (D) Ground plane. (E) Bottom feed layer. (F) Simulated reflection coefficients for both states. (G) Simulated radiation phase for both states. (H) Simulated radiation gains for both states.

Figure 2 illustrates the structure of the mmWave radiation-type metasurface unit. The exploded view of the unit in Fig. 2A reveals that the radiation-type metasurface unit consists of 4 metal layers, 2 dielectric substrate layers, and 1 bonding layer. The 4 metal layers are, in sequence, the radiation patch layer, the bias network layer, the ground plane, and the feed layer. The radiation patch layer is responsible for radiating electromagnetic beams. The bias network layer controls the switching state of the PIN diodes and encodes the unit. The ground plane separates the 2 dielectric layers, ensuring unidirectional radiation of energy. The feed network layer provides power to stimulate the metasurface unit, thus replacing an external feed source. The dielectric material for the radiating layer is Rogers RT5880, which is selected for its excellent high-frequency performance, with a thickness of 0.508 mm and a dielectric constant of 2.2. The backside dielectric material of the feed network layer is Rogers RO4003C, with a thickness of 0.305 mm and a dielectric constant of 3.55. The intermediate bonding layer is Rogers RO4450F, with a thickness of 0.101 mm and a dielectric constant of 3.52, primarily used to bond the 2 dielectric layers, thus forming an integral unit. The total thickness of the metasurface unit is only 0.914 mm, which is consistent with the thickness of the fabricated metasurface array, allowing the entire metasurface system profile to remain within 0.1*λ*, which is significantly smaller than that of conventional metasurfaces with external feeding sources.

Figure 2B to E provide a detailed presentation of the 4 crucial layers of the metasurface unit. The specific parameters of the 4 layers of the metasurface are provided in Table S1. The front radiation layer consists of an elliptical patch, a slot, and 2 PIN diodes integrated into patches extending from the center of the elliptical ring. When one PIN diode is forward-biased and the other is reverse-biased, a wideband dual-resonant U-slot radiation-type metasurface unit can thus be constructed. Metallic vias symmetrically placed on either side of the patch are connected to the bias network layer and interfacing with the FPGA. The FPGA applies a positive or negative voltage to control the switching of the PIN diodes. To suppress high-frequency leakage in the bias network, the bias layer employs narrow-width bias lines, symmetrically distributed meandered inductive lines, and fan-shaped distributed capacitors, thus forming an equivalent RLC circuit that effectively isolates the alternating-current effects from influencing the PIN diode switching. The ground plane could mitigate the useless backward radiation as well as prevent energy leakage. The feed network, located on the reverse side of the ground plane, connects to the radiation patch layer through a central metallized via-hole to enable energy transfer. Additionally, a grounding line extending through the microstrip serves as a dc block, thus enabling harmonic suppression for high-speed control signals.

In the radiation patch layer, 2 PIN diodes (MACOM MADP-00907-14020) are integrated, with their switching controlled by an FPGA, to enable the unit to toggle between 0 and π states, thus achieving programmable functionality. When a forward bias is applied, the PIN diode is modeled as a series combination of a resistor and an inductor (*R* = 5.2 Ω and *L* = 30 pH). Under reverse bias, it behaves as a series combination of a capacitor and an inductor (*C* = 0.025 pF and *L* = 30 pH). This equivalent diode model allows the metasurface unit to switch between 2 states (0 and π). Compared to traditional passive metasurfaces, this active metasurface with PIN diodes allows real-time encoding and adjustment for various application scenarios.

The numerical simulation of the mmWave radiation-type programmable metasurface unit was obtained by using the professional electromagnetic simulation software CST Microwave Studio. A 90-Ω discrete port is placed at the end of the feed line, and simulations are performed using a time-domain solver with periodic boundary conditions. During the simulation process, the unit results were derived by employing discrete-port excitation under a time-domain solver. Figure 2F to H present the simulation results of the metasurface unit under 2 different coding states. Specifically, as shown in Fig. 2F, the *S*_11_ parameter remains below −10 dB within the frequency band of 27.68 to 30.62 GHz, achieving a relative bandwidth of over 10%. The radiation phase of the unit, depicted in Fig. 2G, reveals that the phase difference between the 2 states is approximately 180° at a center frequency of 29 GHz, demonstrating its capability of achieving 1-bit resolution with high stability. Finally, as shown in Fig. 2H, the radiation gains for the 2 coding states are 6.50 and 6.63 dBi, respectively, indicating excellent performance that satisfies the requirements for high-gain and low-side-lobe array design.

### Conformal metasurface array design

Based on the proposed metasurface unit, a mmWave conformal radiation-type programmable metasurface array is designed, as illustrated in Fig. S1. The metasurface array is designed with a cross-shaped configuration, which is composed of 260 units and arranged in an 18 × 18 grid with four 4 × 4 subarrays subtracted at the corners. The overall effective size is 8*λ*_0_ × 8*λ*_0_, where *λ*_0_ is the free-space wavelength at the middle frequency. The purpose of the cross-shaped conformal design is to seamlessly integrate with the antenna radome of communication satellites, thus meeting the system requirements for lightweight design and efficient space utilization. In addition, the cross-shaped metasurface features circular chamfers around its edges, with a certain reserved width between the metal frame, which effectively reduces the issue of uneven phase distribution at the edges.

A total of 520 PIN diodes are soldered on top of the metasurface to provide individual phase control via a biasing network. The biasing network layer of the metasurface is divided into 8 segments and connected with the FPGA through flexible printed circuit connectors, with each segment corresponding to 32 or 33 units. The biasing network is symmetrically designed in 4 pairs of sections to simplify wiring and minimize interference in the biasing circuit. To excite the metasurface units, a hybrid serial–parallel microstrip network composed of unequal power dividers is used. This network is integrated within the metasurface array, reducing the overall system profile. Chebyshev weighting is applied to the network, with the amplitude at each stage of the feed network satisfying the formula [43]

$$\begin{array}{c} I_{n}=\sum_{q=n}^{M}{\left(-1\right)}^{M-q}\frac{x_{0}^{2q-1}\left(q+M-2\right)!\left(2M-1\right)}{\left(q-n\right)!\left(q-n+1\right)!\left(M-q\right)!},\\ \;n=1,2,\ldots ,M \end{array}$$ (1)

where *q* represents the polynomial order. Through the formula, the Chebyshev weighting coefficients for each stage of the feed network are calculated and combined with the basic impedance transformation concept, and the length and width parameters of the microstrip lines at each stage can thus be determined. The introduction of Chebyshev weighting effectively enhances the main-lobe gain and reduces the side-lobe levels. Furthermore, the feed network can introduce an initial phase gradient to prevent side lobes of an equivalent network during beam steering with a 1-bit phase resolution. The symmetrical design of the feed network could avoid field distribution disruption on the metasurface, thus maintaining low side-lobe levels.

Figure S2 illustrates the initial phase distribution of the metasurface. Electromagnetic waves propagate radially from the center through the integrated hybrid microstrip network, gradually feeding the top metasurface units with an initial weighted phase distribution to reduce the side-lobe levels. The initial phase provided by the feed network beneath the metasurface could be obtained when all units are set to the same programmable state.

### OAM beaming generation

To achieve the focused excitation of mmWave OAM beams by using a radiation-type metasurface, the optimal phase distribution of the metasurface needs to be adjusted according to the following constraint equation:

$$\phi_{\mathrm{OAM}}\left(x,\;y\right)=l\cdot \text{arctan}\left(y/x\right)+\phi_{0}$$ (2)

where (*x*, *y*) represents the relative coordinates of each unit and *ϕ*_0_ denotes the initial phase as shown in Fig. S2. The phase variation can further be quantified using the following 2 expressions:

$$\begin{array}{c} \phi_{\text{QOAM}}=\left\{\begin{array}{cc} 0, & \;\phi_{\mathrm{OAM}}\in \left[0+2\mathrm{n\pi},\pi +2\mathrm{n\pi}\right)\\ \pi , & \;\phi_{\mathrm{OAM}}\in \left[\pi +2\mathrm{n\pi},2\pi +2\mathrm{n\pi}\right) \end{array}\;n\in Z\right. \end{array}$$ (3)

**Fig. 3.**
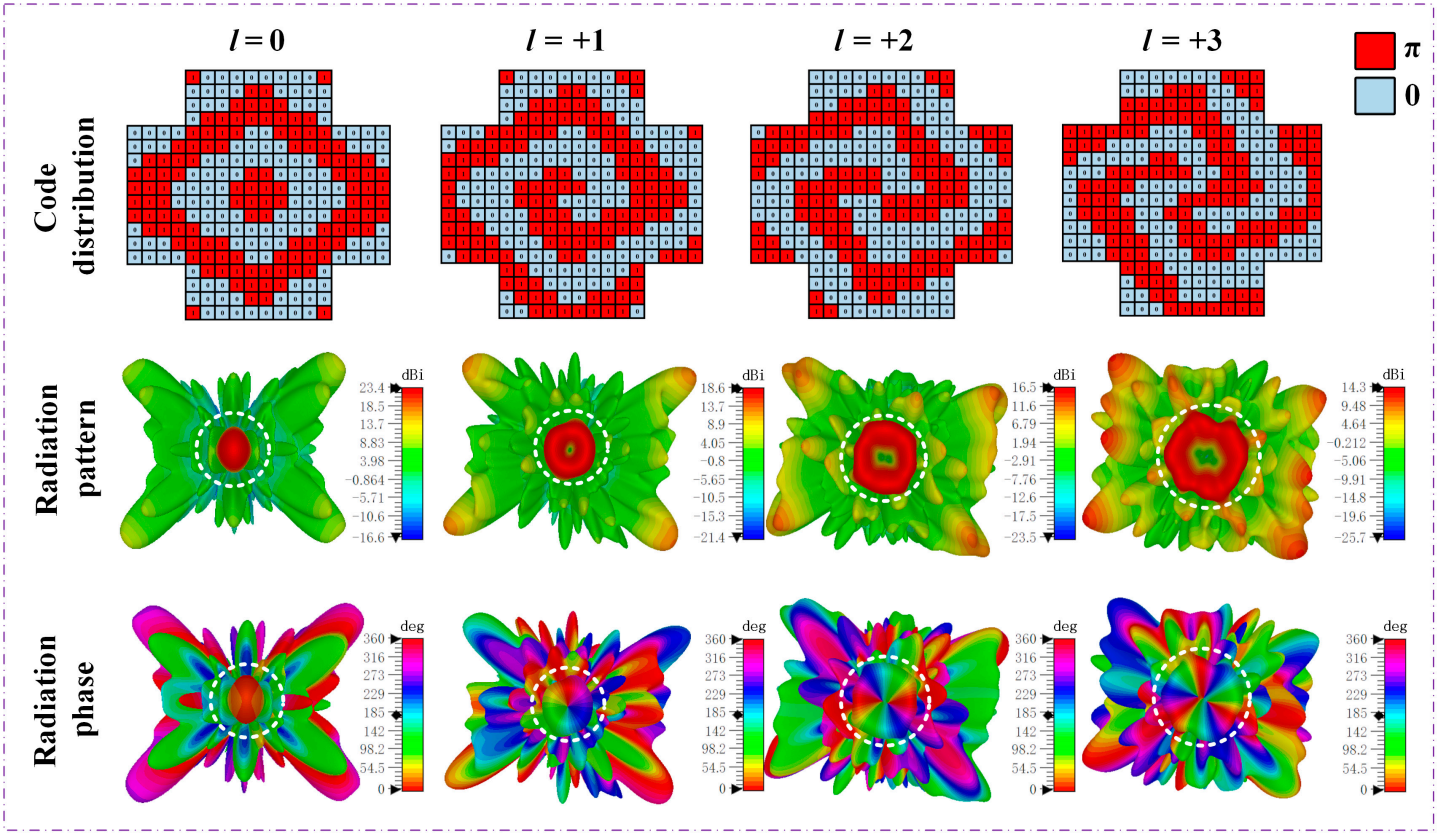
Code distributions and simulated radiation and phase patterns for different OAM modes.

To validate the proposed radiation-type metasurface for dynamic OAM beam generation, simulations were conducted for 4 OAM modes at the center frequency, namely, *l* = 0, *l* = +1, *l* = +2, and *l* = +3. The quantized coding of the metasurface for these 4 OAM modes and their corresponding 3-dimensional directional radiation and phase patterns are illustrated in Fig. 3. The OAM wave coding for each mode is derived from Eq. 3 and shown in the first column. The far-field radiation amplitude patterns for the 4 OAM modes are displayed in the second column, clearly revealing the strong vortex-shaped radiation. The far-field phase patterns of the 4 OAM modes are presented in the third column, exhibiting typical OAM phase characteristics, with phase singularities clearly observed along the metasurface axis. For the *l* = +1 mode, the phase variation of the electromagnetic wave over one cycle is 360°. For *l* = +2, it is 720°. Furthermore, for *l* = +3, the phase variation reaches 1,080°. For the negative modes *l* = −1, *l* = −2, and *l* = −3, based on the conventional mirror principle, the phase variation is similar to that of the positive modes, but the direction reverses from clockwise to counterclockwise. Thus, high-purity OAM beams were successfully generated for the 4 modes by using the designed metasurface; the purity of different OAM beams can be seen in Fig. S3.

Since a single-directional OAM wave may not adequately meet the needs of scenarios involving a moving target, the commonly used beam scanning technique can thus be combined with OAM wave excitation to realize OAM wave scanning. To steer the beam in a specific direction, the optimal phase distribution of the metasurface must satisfy the following equation:

$$\begin{array}{c} \phi_{\text{SCAN}}\left(x,\;y\right)=k_{0}\left(x\text{sin}\theta \text{cos}\varphi +y\text{sin}\theta \text{sin}\varphi \right)+\phi_{0} \end{array}$$ (4)

where $\left(\theta ,\varphi \right)$ represents the designed scanning direction and (*x*, *y*) denotes the relative coordinates of each unit. By combining the phase of Eq. 4 with Eq. 2 based on the convolution operations through coding a metasurface [44], the single-directional OAM wave can then be scanned across different directions. Since the designed metasurface has a 1-bit phase resolution, further quantization of the combined phase expression can be obtained as follows:

$$\begin{array}{c} \begin{array}{c} \phi_{\text{SCAN}\_\mathrm{OAM}}=\left\{\begin{array}{cc} 0, & \;\phi_{\mathrm{OAM}}+\phi_{\text{SCAN}}\in \left[0+2\mathrm{n\pi},\pi +2\mathrm{n\pi}\right)\\ \pi , & \;\phi_{\mathrm{OAM}}+\phi_{\text{SCAN}}\in \left[\pi +2\mathrm{n\pi},2\pi +2\mathrm{n\pi}\right) \end{array}\;n\in Z\right. \end{array} \end{array}$$ (5)

**Fig. 4.**
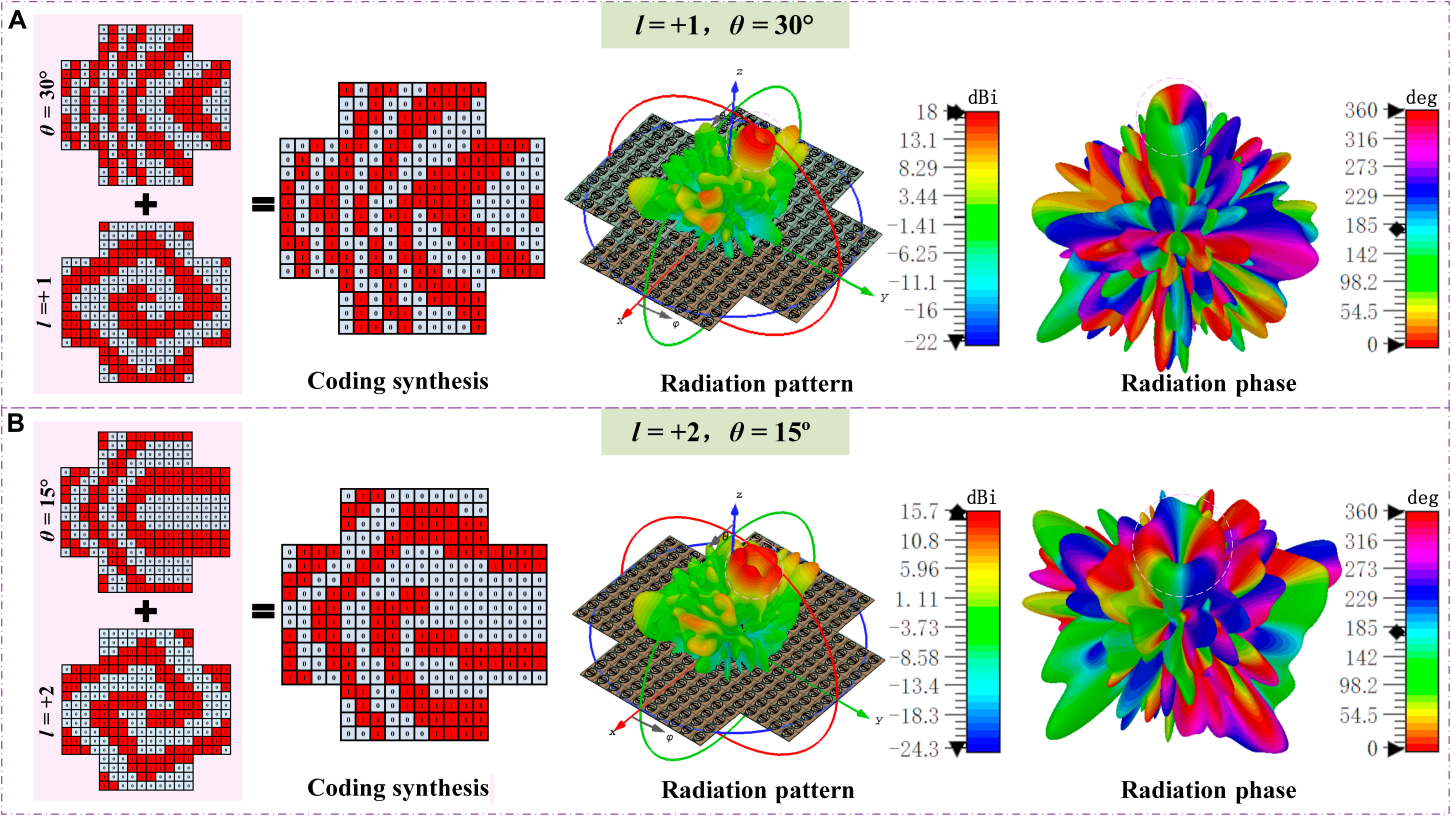
Code distributions and simulated radiation patterns. (A) OAM mode *l* = +1 with scanning angle *θ* = 30°. (B) OAM mode *l* = +2 with scanning angle *θ* = 15°.

To validate the proposed radiation-type programmable metasurface for OAM wave beam scanning, the OAM and beam scanning codes were combined, and each unit was controlled by using an FPGA. An OAM mode *l* = +1 with a 30° beam deflection and an *l* = +2 OAM wave with a 15° beam deflection are generated respectively, as shown in Fig. 4. The left component of Fig. 4 clearly demonstrates the superposition of phase coding, where the individual codes for both applications were computed, synthesized, and quantized into binary states to achieve OAM beam scanning at different angles. Figure 4A illustrates the OAM beam with an *l* = +1 mode and a 30° scanning angle, where the vortex center is shifted by 30°, accompanied by a 360° phase rotation. Figure 4B depicts the OAM beam with an *l* = +2 mode and a 15° scanning angle, with the vortex center shifted by 15° and accompanied by a 720° phase rotation. More OAM beam scanning simulation results can be found in Fig. S4. This beam scanning of OAM waves effectively addresses scenarios with moving objects, ensuring continuous reception of OAM waves. It holds significant potential in future secure communications and holographic imaging applications [13,45].

### Experimental verification

**Fig. 5.**
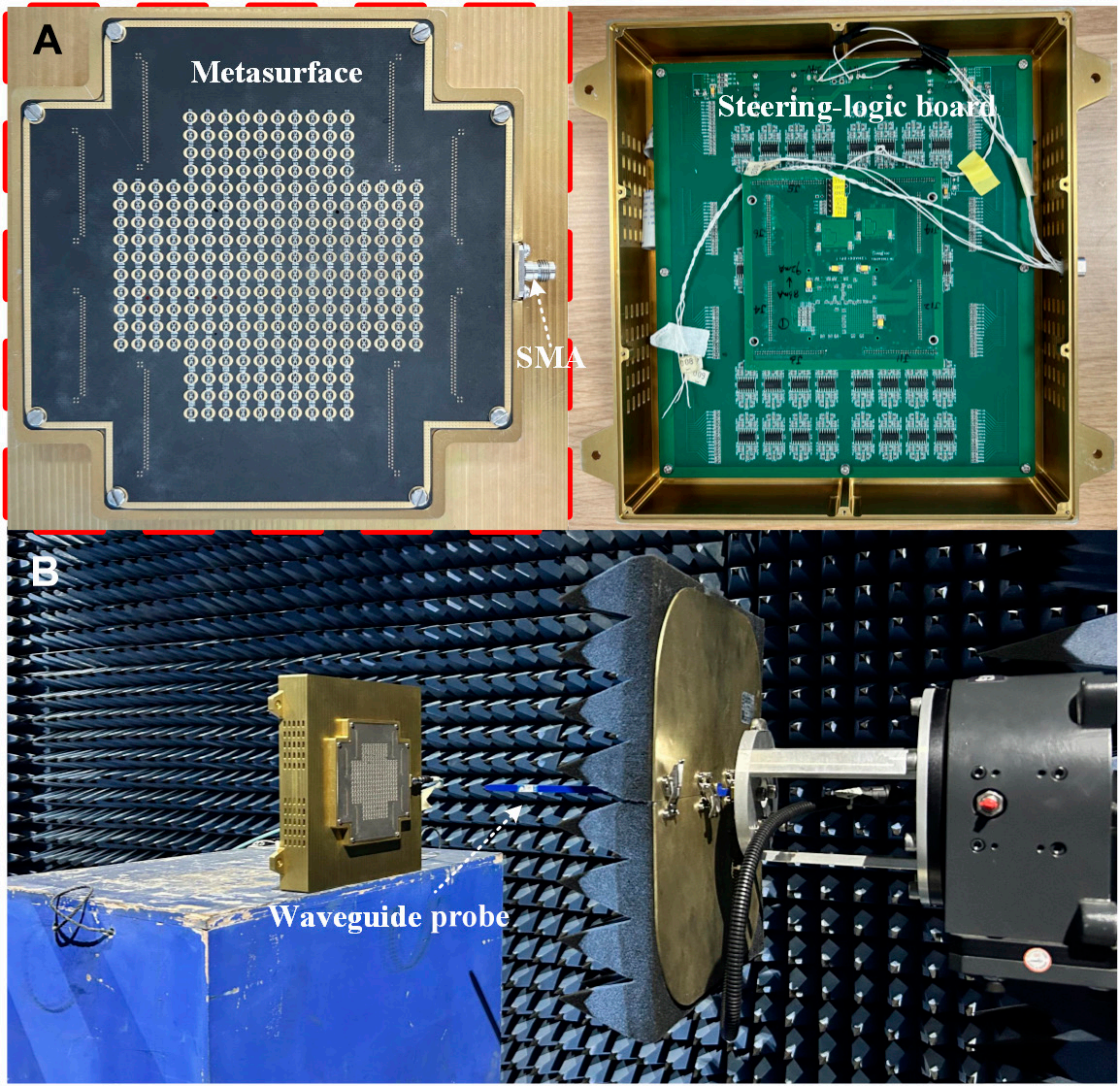
Physical prototype of the proposed metasurface. (A) Metasurface with a metallic frame and SMA connectors, along with a steering-logic board. (B) Near-field testing experiments of the metasurface conducted in an anechoic chamber environment.

To further validate the actual OAM wave generation capability of the conformal radiation-type programmable metasurface at the mmWave band, we fabricated a cross-shaped metasurface comprising 260 units, and each unit was equipped with 2 PIN diodes for switching between 2 operational states, as shown in Fig. 5A. The units were fed by a series–parallel hybrid feeding network integrated within the metasurface, with SMA connectors soldered at the network’s terminal to interface with radio-frequency signals. Additionally, a square metallic frame was designed on the back of the metasurface for integration of a steering-logic board. The control board is based on a Xilinx Kintex-7 FPGA, capable of outputting 400 signals, 260 of which were used for controlling the metasurface units, while the remaining ports were grounded. The FPGA was powered by a 24-V current source, with parallel clock cycle signals controlling the bias network to enable real-time coding and modulation of each unit. The measured voltages across the PIN diodes in the 0 and π states were −1.3 and +1.3 V, respectively, with a current of 10 mA per unit. Consequently, the total power consumption of the metasurface was only 3.38 W, demonstrating low-power and high-efficiency characteristics.

The mmWave conformal radiation-type programmable metasurface was tested in a near-field anechoic chamber, as shown in Fig. 5B. An open-ended rectangular waveguide probe was used to scan the effective area of the metasurface and receive the radiated electromagnetic waves, and the sampling waveguide was positioned approximately 3 wavelengths from the metasurface. The sampling region was divided into 45 × 45 subregions, which were sampled by the rectangular waveguide to obtain the radiation characteristics under different coding schemes. To acquire the amplitude and phase data of the near field, the metasurface was connected to a vector network analyzer (Keysight N5222a) via an SMA connector. During testing, the metasurface remained stationary while the waveguide probe moved in a serpentine pattern along the *x* and *y* directions, thus resulting in a complete OAM near-field pattern across the entire area.

The experimental *S*_11_ results of the metasurface array obtained in the anechoic chamber, along with the specific experimental data, are presented in Fig. S5 and Table S2, respectively. There is a certain discrepancy between experimental data and simulation results. This may be due to manufacturing errors caused by the small size, impedance mismatching at the junctions, or inconsistencies between the boundary conditions in the simulation software and the testing environment. The metasurface could cover the operating frequency band from 27.38 to 30.75 GHz, with *S*_11_ remaining below −10 dB in both simulation and experiment within this range, indicating favorable wideband characteristics.

**Fig. 6.**
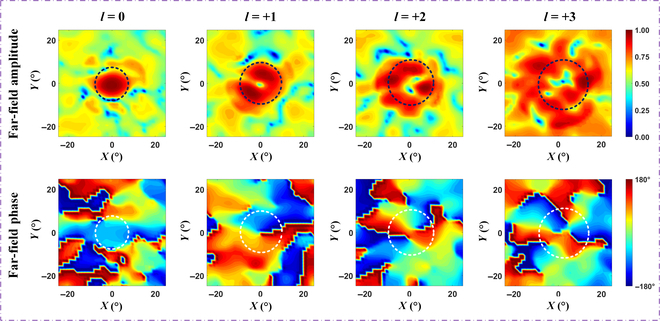
Experimental normalized far-field amplitude and phase results of the metasurface for the 4 OAM modes *l* = 0, *l* = +1, *l* = +2, and *l* = +3.

Figure 6 illustrates the measured far-field amplitude and phase distributions for the 4 OAM modes *l* = 0, *l* = +1, *l* = +2, and *l* = +3. As the mode number increases, the nonradiative area of the OAM beam expands. The phase singularities of the characteristic vortex wavefront along the axis also demonstrate the effectiveness of the proposed metasurface for generating nonzero OAM modes. Measurement results indicate a certain degree of distortion in the generated OAM beams, particularly at higher modes. This distortion may be attributed to the very high frequency, the relatively small size of the metasurface unit, and fabrication errors that occurred during the process of soldering SMA connectors to the feeding network. Another contributing factor may be the limited binary phase resolution of the proposed 1-bit reconfigurable unit, where continuous phase variation is typically required. By improving phase accuracy, such as upgrading from 1 bit to higher bits or increasing the metasurface array size, the distortion of OAM waves can be effectively reduced. Under experimental conditions, the proposed mmWave metasurface could obtain a peak gain of 22.54 dBi at a center frequency of 29 GHz, with an overall aperture efficiency of 21.75%, which was calculated by the following equation:

$$\;\eta =\frac{G\lambda^{2}}{4\pi A}$$ (6)

where *A* represents the area of the metasurface array and *G* represents the peak gain of the metasurface at a frequency of 29 GHz. Although the aperture efficiency is only 21.75%, it is influenced by several factors, including limited phase resolution, dielectric losses, and manufacturing errors. Additionally, to achieve conformal integration, the metasurface array is designed in a cross-shaped configuration, sacrificing 64 units compared to a square array. However, this trade-off may result in higher integration and practicality.

**Fig. 7.**
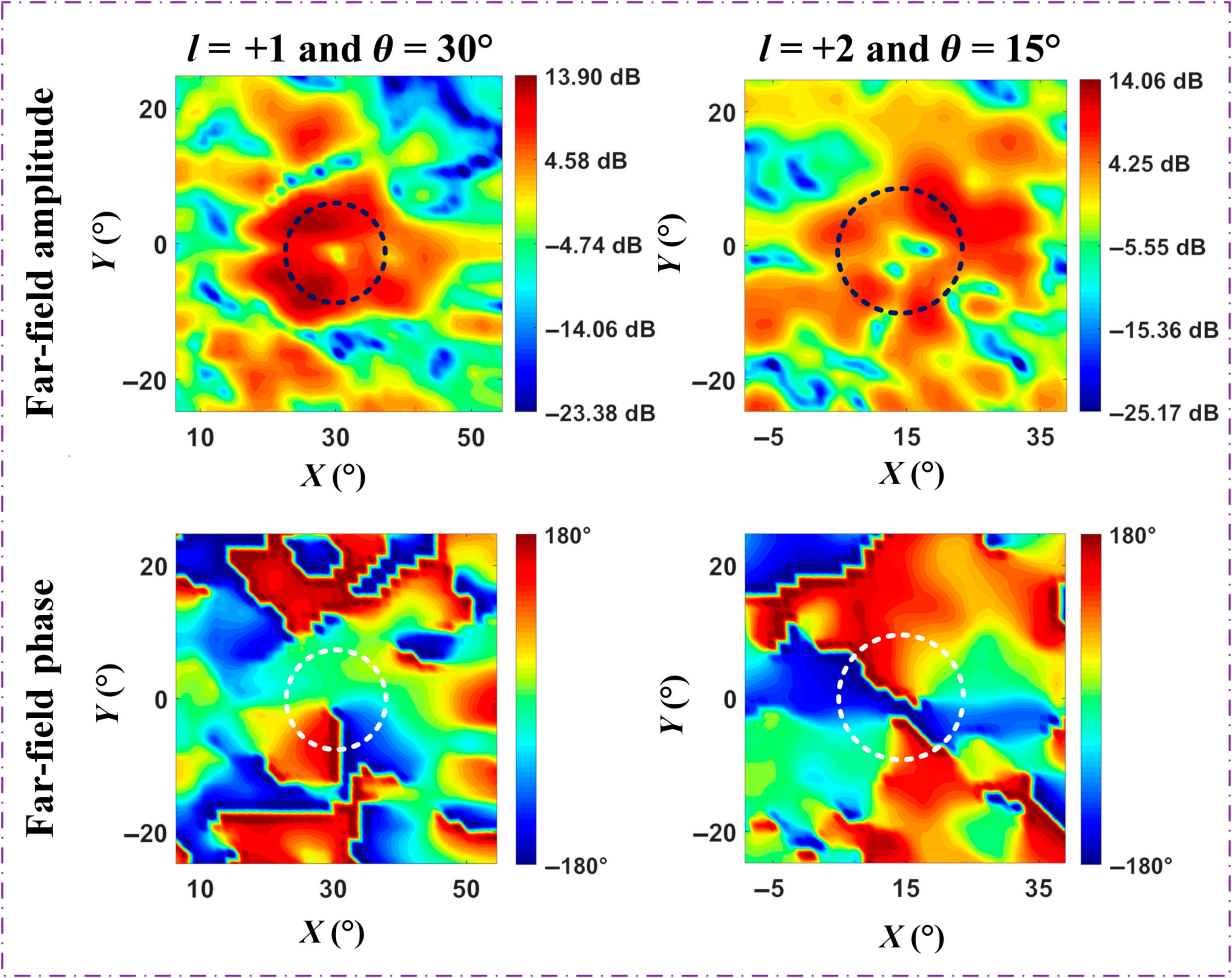
Experimental far-field amplitude and phase results of the OAM wave for mode *l* = +1 with scanning angle *θ* = 30° and mode *l* = +2 with scanning angle *θ* = 15°.

Figure 7 displays the experimental results of OAM waves *l* = +1 and *l* = +2 at scanning angles of 30° and 15°, respectively. For the case of OAM mode *l* = +1, a 360° phase variation is observed, while OAM mode *l* = +2 demonstrates a 720° phase variation, consistent with the expected helical phase characteristics. More OAM beam scanning experimental results can be found in Fig. S6. The experimental results indicate that the OAM beams could maintain stable structural properties across different scanning angles, which exhibit good directivity along with a clear phase distribution, thereby verifying the stability and reliability of our proposed design. The proposed programmable metasurface shows potential in next-generation communication systems, but it still faces challenges such as manufacturing precision for mmWave or higher frequencies, scalability for larger arrays, and the impact of the complex space environment on the metasurface reliability. Therefore, exploring alternative reconfigurable strategies is crucial. A promising approach is to use optical or electrical pulses to dynamically adjust the properties of the metasurface, which can further optimize the metasurface design to meet the demands of future communication systems [46]. In the future, the designed metasurface is expected to scale up to larger arrays and higher frequencies, but the complexity of thermal management and feed network will increase accordingly.

## Conclusion

In summary, a conformal radiation-type programmable metasurface has been designed to excite multimode mmWave OAM beams and further conduct OAM beam scanning. By utilizing a feeding network instead of the traditional external spatial feed, the overall profile of the metasurface system is significantly reduced. Active PIN diodes are introduced to modulate the resonant characteristics of the metasurface unit and realize stable phase states and favorable radiation properties. A cross-shaped programmable metasurface with 260 units has been designed and fabricated, and its application for generating high-purity multimode mmWave OAM beams has been verified both numerically and experimentally. The proposed conformal cross-shaped architecture is ultraportable and very effective in integrating with the front ends of satellites or aircraft and eliminating issues such as feed source blockage and energy spillover losses in conventional metasurfaces. The metasurface could obtain a wide operation bandwidth of over 10% and a measured gain of 22.54 dBi with an aperture efficiency of over 21.75% while successfully exciting 4 high-purity OAM wave modes, namely, *l* = 0, *l* = +1, *l* = +2, and *l* = +3. Additionally, by incorporating beam scanning techniques, OAM waves could be deflected to accommodate scenarios with moving receivers, demonstrating significant potential for next-generation wireless communications and future space-based satellite applications.

## Methods

Details about the methods used for this research in both simulation and measurement are provided in the Supplementary Materials.

## Data Availability

The data that support the findings of this study are available from the corresponding authors upon reasonable request.
