# Supplementary material for: Conformal Radiation-Type Programmable Metasurface for Agile Millimeter-Wave Orbital Angular Momentum Generation
Source: Research (Wash D C). 2025 Mar 14;8:0631. doi: 10.34133/research.0631 (PMC11906973; doi:10.34133/research.0631)
Supplement: Supplementary 1 — Tables S1 and S2 Figs. S1 to S6 [file research.0631.f1.docx]

**Supplementary Material for**

Conformal Radiation-type Programmable Metasurface for Agile Millimeter-Wave OAM Generation

**Authors**

Anjie Cao^1^, Tao Ni^1^, Yuhua Chen^2^, Longpan Wang^2^, Zhenfei Li^2^, Xudong Bai^2^*,

Fuli Zhang^2^*, and Zhansheng Chen^1^*

**Affiliations**

^1^ Shanghai Institute of Satellite Engineering, Shanghai 201109, China

^2^ Northwestern Polytechnical University, Xi'an 710129, China

* Correspondence should be addressed to: Zhansheng Chen; zhansheng.chen@ieee.org, 1 Fuli Zhang; [fuli.zhang@nwpu.edu.cn](mailto:fuli.zhang@nwpu.edu.cn) and Xudong Bai; [baixudong@nwpu.edu.cn](mailto:baixudong@nwpu.edu.cn)

**Simulation**

To validate the accuracy of the millimeter-wave (mmWave) unit simulation, **Table S1** lists the key parameters of the radiation-type programmable metasurface unit, corresponding to those marked in Fig. 2 of the main text. The metasurface unit was modeled and simulated by using CST Microwave Studio with a 90Ω discrete port introduced at the end of the unit’s microstrip line for time-domain simulation, thus acquiring the optimized simulation parameters presented in the main text.

***Table SI. Parameters of the conformal radiation-type programmable metasurface (Unit:mm).***


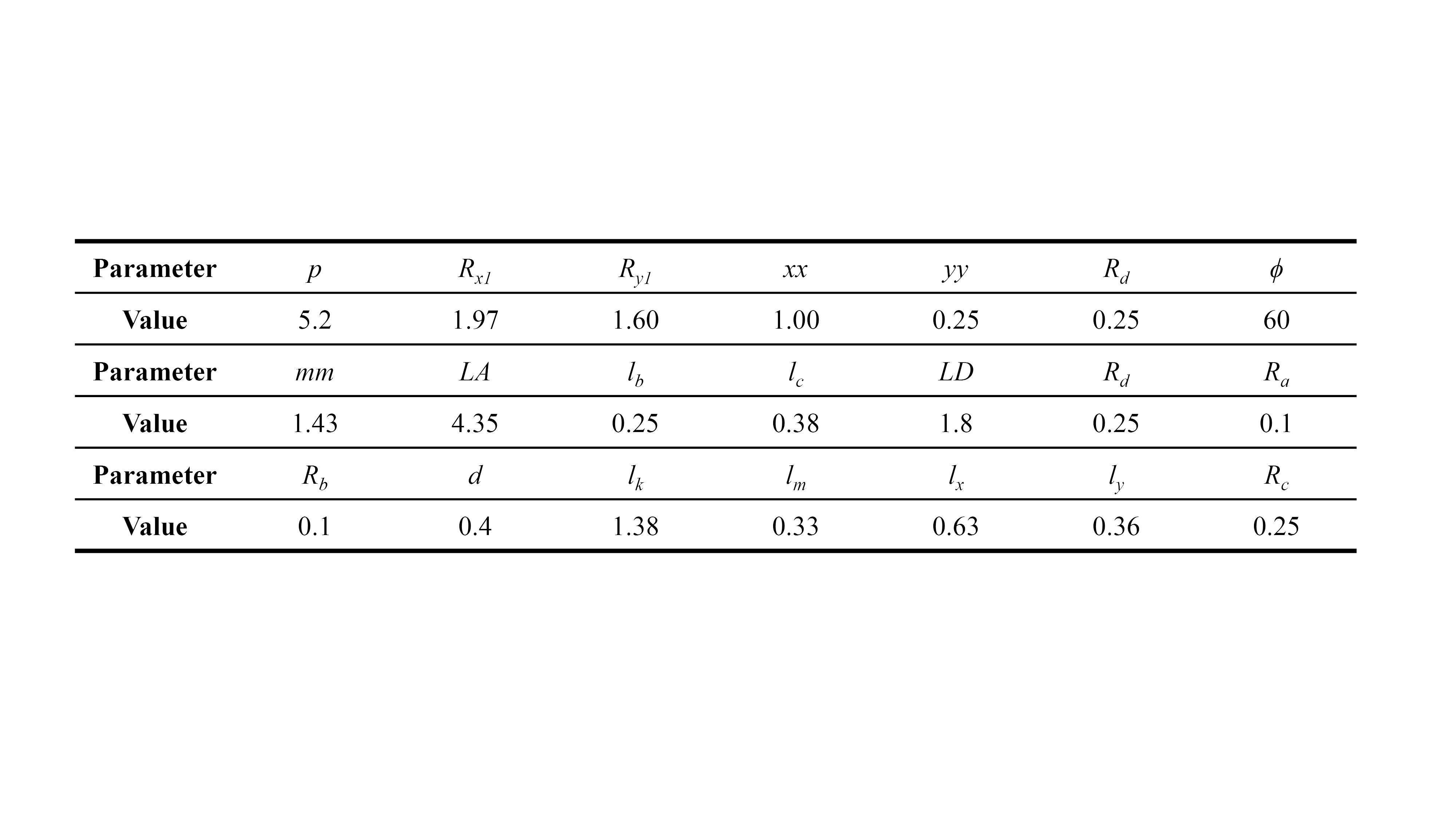


**Simulation**

**Fig. S1**(a) and (b) depict the radiation patch layer and the feed network layer of the metasurface array, which adopt a cross-shaped configuration. This design facilitates better integration with external structures, such as the front of aircraft and satellites, thus enhancing the environmental applicability of the metasurface. The metasurface array comprises 260 units, with two PIN diodes soldered onto each unit to control the phase coding. The feed network adopts a series-parallel hybrid configuration, incorporating impedance transformation and Chebyshev weighting. The feed network is first designed separately as a 1×10 and a 1×18 network in the x-direction and an 18×18network in the y-direction before integrating into the overall array. The integrated feed network could effectively replace conventional external feed sources in conventional metasurfaces, thus reducing spatial energy losses as well as significantly lowering the system profile.


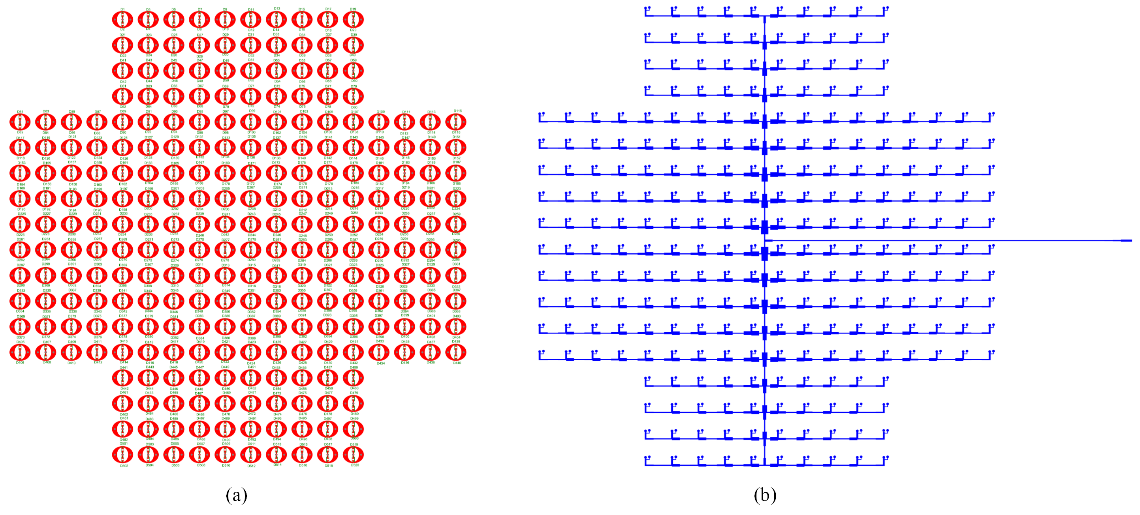


***Fig. S1. Schematic diagram of the conformal mmWave radiation-type programmable metasurface array.*** *(a) Radiating layer, (b) Feeding network layer.*

**Simulation**

**Fig. S2** displays the initial phase distribution of the metasurface at the central frequency of 29 GHz. Due to the varying time delays as RF signals propagate through the feed network, initial phase differences can thus be created, which can be obtained when all units are set to the same programmable state. The initial phase gradient is very effective to prevent sidelobes of an equivalent network during beam steering with a 1-bit phase resolution.


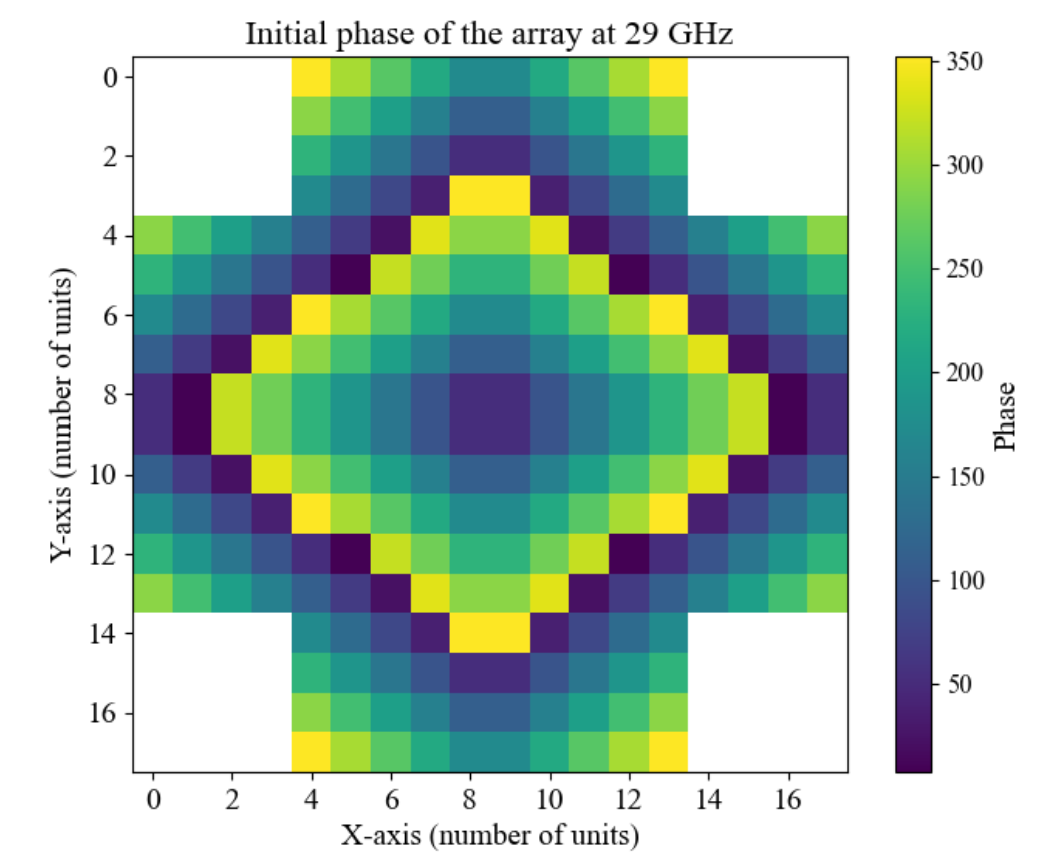


***Fig. S2. Initial phase gradient of the designed metasurface array at 29 GHz.***

**Simulation**

**Fig. S3** presents the normalized bar chart of the purity values of OAM waves under the l = +1, l = +2, and l = +3 modes. It can be observed that in all three modes, the main mode values are significantly more prominent compared to the other component modes, with some contributions from adjacent modes, but these are relatively small. This indicates that the purity values of the three OAM waves generated by the designed metasurface are very high.


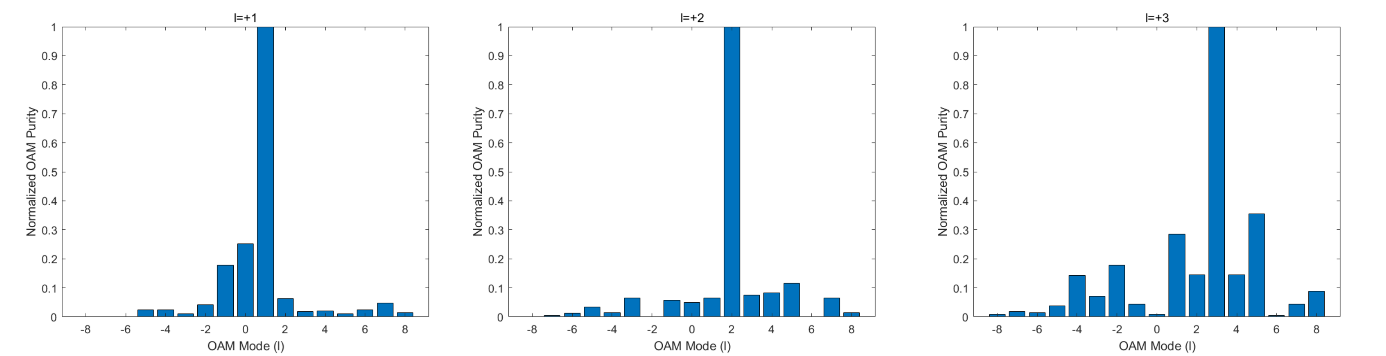


***Fig. S3. Normalized bar chart of the mode purity of OAM waves under three different modes (l = +1, l = +2, l = +3).***

**Simulation**

**Fig. S4** demonstrates the vortex wave beam scanning generated under two additional scenarios: vortex wave generation at *θ* = 15° for the l = +1 mode and vortex wave generation at *θ* = 30° for the l = +2 mode. It can be observed that both cases exhibit favorable results, with distinct vortex-shaped wavefronts pointing toward the corresponding angles, and the phase variation is relatively uniform. The vortex wave scanning at these two angles provides further evidence of the feasibility of the proposed metasurface for realizing such applications.


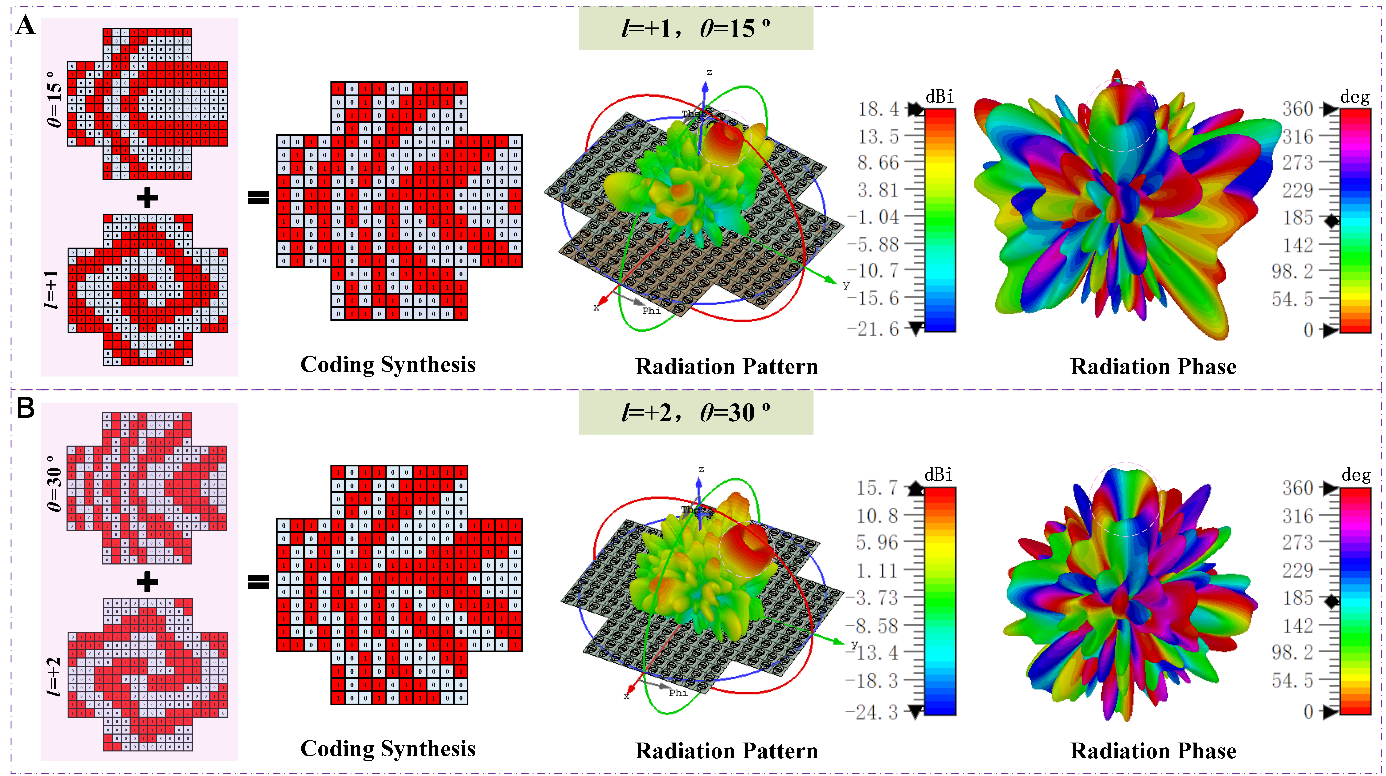


***Fig. S4.*** ***Code distributions and simulated radiation patterns for OAM mode l=+1 with scanning angle θ=15°, and mode l=+2 with scanning angle θ=30°.***

**Measurements**

**Fig. S5** presents the measured and simulated reflection coefficient (S_11_) for the metasurface. Both results cover the operating frequency band from 27.38 GHz to 30.75 GHz for S11<-10dB. Due to the manufacturing tolerances and assembly errors, the experimental result shows some variations with the simulated one, but still remains within the target operating band, indicating favorable wideband characteristics. The experimental results verify the feasibility of the simulation.





***Fig. S5. S_11_ parameters of programmable metasurface obtained from simulation and experimental results.***

**Measurements**

**Table. S2** presents the S_11_ data results under different frequencies for both simulation and testing scenarios. Some discrepancies are observed between the measured and simulated results, which may be attributed to the following reasons: 1) At millimeter-wave frequencies, the small size of the metasurface makes it highly sensitive to manufacturing errors, leading to variations in phase and amplitude characteristics. 2) Simulations typically assume ideal open boundary conditions, while in experiments, actual boundaries may introduce electromagnetic wave reflections. 3) In the experiment, the solder joints between the feed network and SMA connectors may introduce additional losses or mismatches, affecting the accuracy of the reflection coefficient.


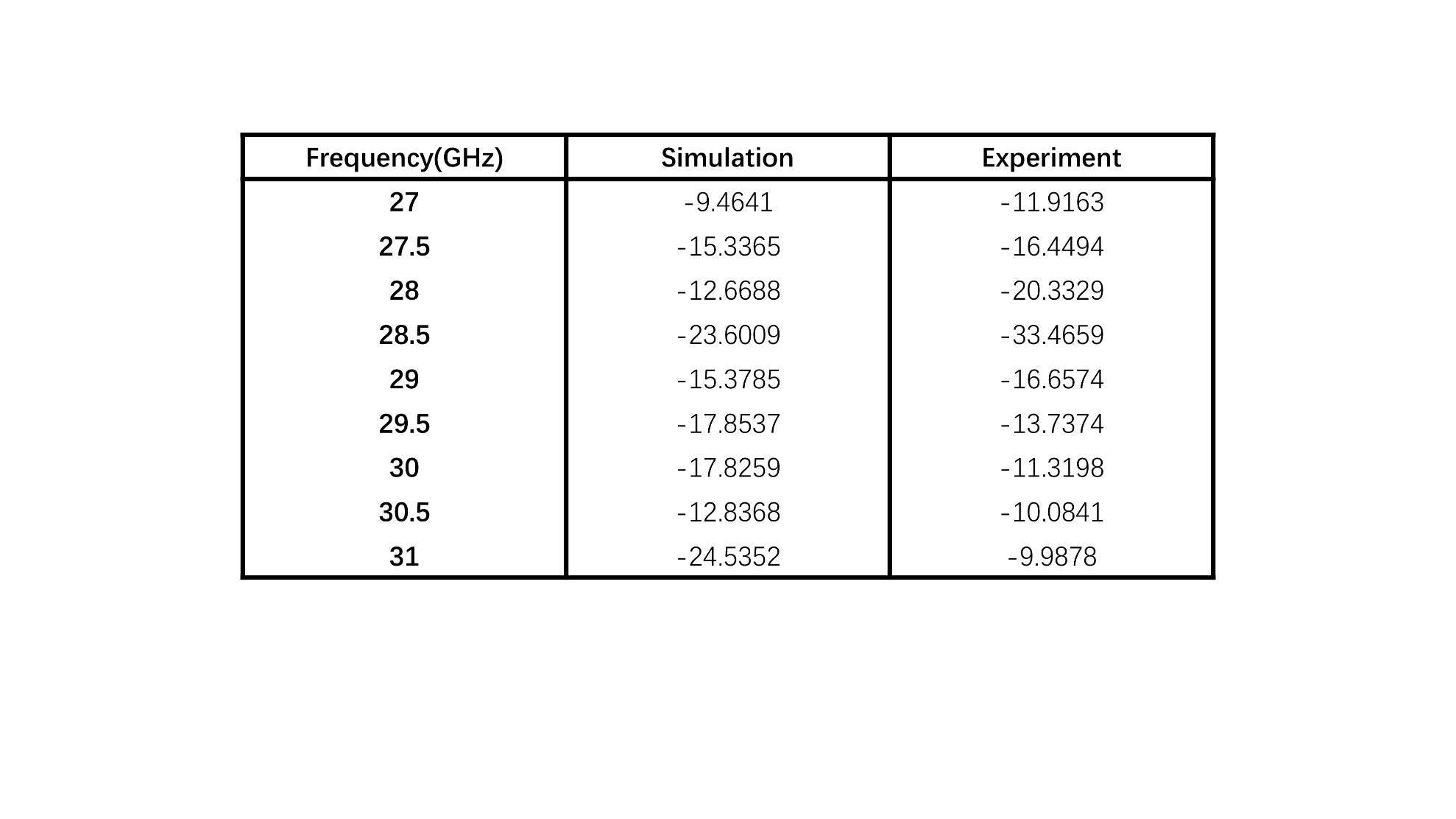


***Table. S2. Specific Comparison of S_11_ Simulation and Testing Data.***

**Measurements**

**Fig. S6** displays the far-field gain and phase for the beam scanning at 15° under the l = +1 mode and at 30° under the l = +2 mode. It can be observed that, due to the higher mode and larger scanning angle, the phase variation is less continuous in the second case. However, overall, the vortex wavefront images remain clear, indicating that the designed metasurface is capable of realizing OAM beam scanning, which holds significant potential for next-generation wireless communication applications.


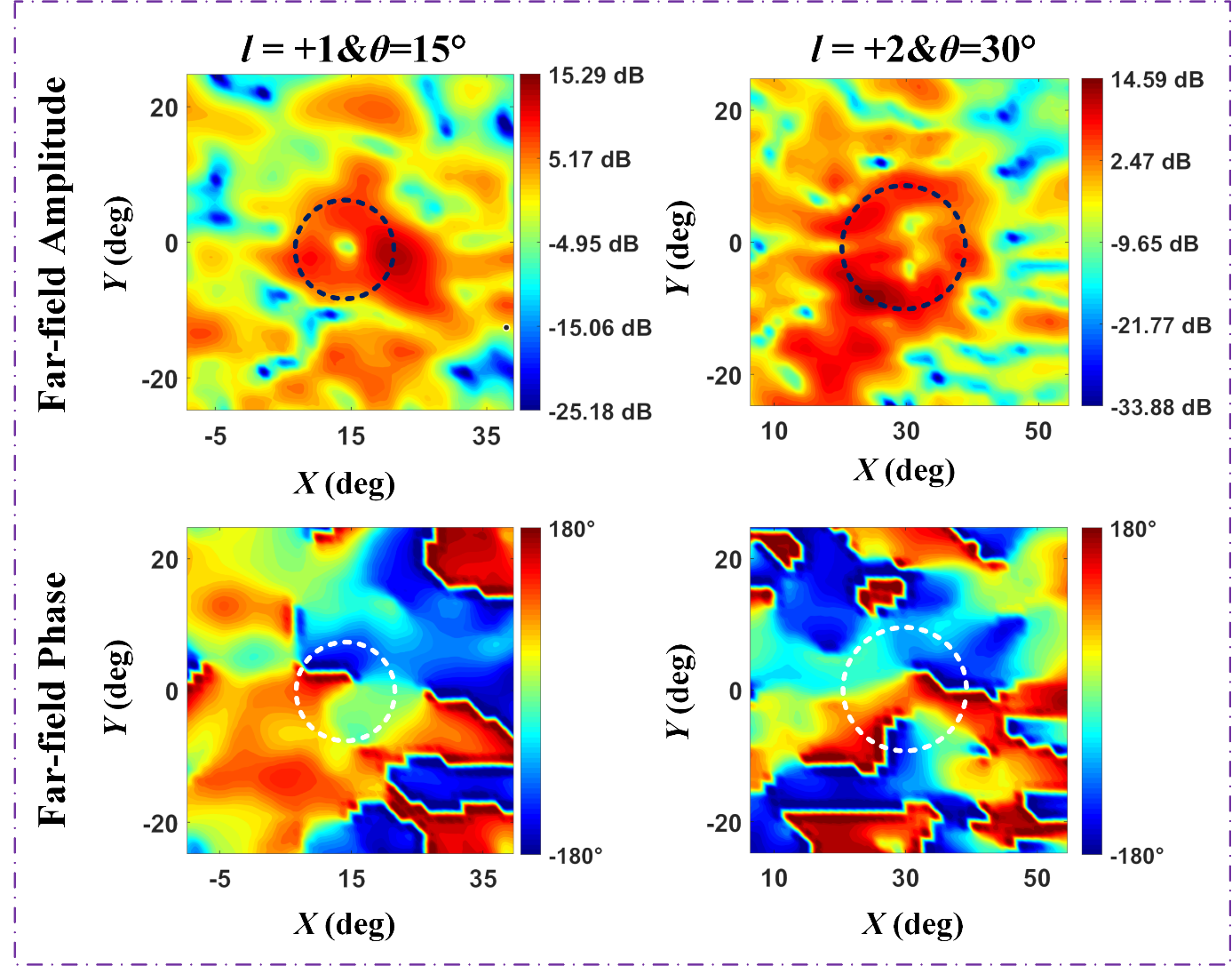


***Fig. S6. Experimental far-field amplitude and phase results of the OAM wave for mode l=+1 with scanning angle θ=15°, and mode l=+2 with scanning angle θ=30°.***
